# Enhancing the Resistance Welding of GF/PEI Composites via a PECVD-Deposited HMDSO Coating on a Heating Element

**DOI:** 10.1021/acsomega.6c00031

**Published:** 2026-04-13

**Authors:** Luís Felipe Barbosa Marques, Jonas Frank Reis, Emanuele Schneider Callisaya, Felipe Vicente de Paula Kodaira, Edson Cocchieri Botelho, Rogério Pinto Mota, Luis Rogério de Oliveira Hein

**Affiliations:** † São Paulo State University (UNESP), School of Engineering and Science, Department of Materials and Technology, av. Dr. Ariberto Pereira da Cunha, 333 - Pedregulho, Guaratinguetá, São Paulo 12516-410, Brazil; ‡ São Paulo State University (UNESP), School of Engineering and Science, Department of Physics, av. Dr. Ariberto Pereira da Cunha, 333 - Pedregulho, Guaratinguetá, São Paulo 12516-410, Brazil

## Abstract

Resistance welding of thermoplastic composites critically depends on the quality of the metal–polymer interface formed between the heating element and the polymer matrix. However, the intrinsic chemical incompatibility between metallic heating elements and high-performance thermoplastics often limits joint strength and fracture resistance. In this study, we investigate the use of plasma-engineered siloxane coatings to tailor the interfacial properties of AISI 304 stainless-steel heating elements and enhance the performance of resistance-welded glass fiber/poly­(ether imide) (GF/PEI) joints. Thin films based on hexamethyldisiloxane (HMDSO) were deposited by plasma-enhanced chemical vapor deposition (PECVD), followed by oxygen plasma post-treatment to promote surface functionalization via the formation of polar groups. The modified interfaces were characterized by contact angle measurements, FTIR, XPS, and atomic force microscopy, revealing a transition from hydrophobic to highly polar surfaces driven by the incorporation of oxygen-containing species. Mechanical performance was evaluated by single lap shear strength (LSS) testing combined with detailed fractographic analyses using optical microscopy and scanning electron microscopy. The plasma-engineered interfaces exhibited an increase of approximately 48% in lap shear strength compared to untreated joints, accompanied by a clear transition in fracture mode from interfacial failure to intralaminar and mesh wire rupture mechanisms. These results demonstrate that plasma-deposited siloxane coatings provide an effective strategy to bridge the chemical gap in metal–polymer interactions, offering a versatile route for the design of high-performance welded interfaces in structural composite applications.

## Graphical abstract

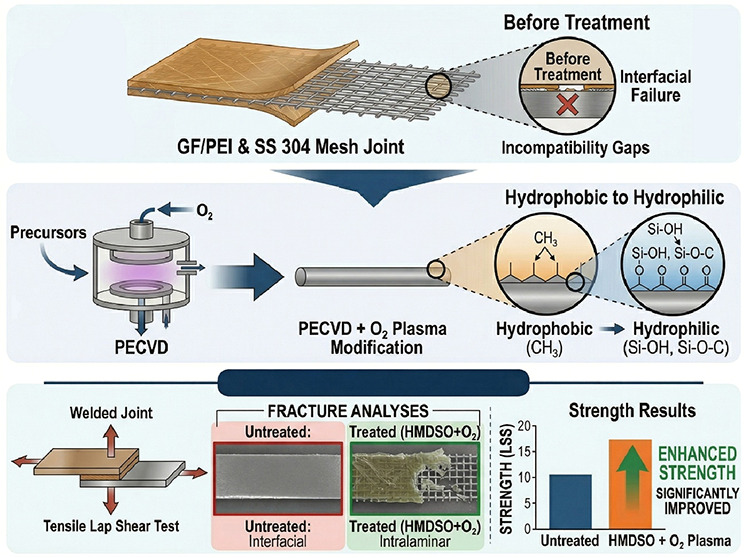

## 1 Introduction

In recent decades, the aerospace industry has progressively replaced metallic alloys with advanced polymeric composites, driven by the need for weight reduction and increased energy efficiency.^1–5^ Among these materials, thermoplastic matrix composites, such as glass fiber-reinforced poly­(ether imide) (GF/PEI), have gained prominence mainly due to their high fracture toughness, excellent chemical resistance, recyclability, and suitability for several joining techniques. Notably, thermoplastic welding techniques are particularly advantageous as they eliminate the need for mechanical fasteners or adhesives, which are typically associated with stress concentrations and long curing cycles, respectively.^2,4,6–8^

The effective joining of these components is critical for structural integrity. Resistance Welding (RW) stands out as one of the most promising fusion joining methods for thermoplastic composites, alongside induction and ultrasonic welding, offering rapid processing cycles and high joint quality.^8–11^ However, this process relies on a heating element (HE), typically a stainless-steel mesh or carbon fibers, which remains embedded at the weld interface after cooling.^12,13^ Although metallic mesh HEs provide efficient heat generation via the Joule effect, the metal and the polymeric matrix exhibit distinct and incompatible chemical natures. Consequently, the HE can act as a defect or facilitate crack propagation, potentially resulting in poor interfacial adhesion and limiting the ultimate mechanical efficiency of the joint.^14–16^

To overcome these challenges, surface modifications of the interfacial contact elements have been consistently explored as potential solutions.^2,4,17–21^ Treatment of the metallic surface is crucial for promoting chemical and mechanical adhesion mechanisms with the polymer matrix.^22–25^ Among the various available techniques, plasma treatments emerge as an attractive solution due to their ability to integrate multiple steps into a single, more environmentally friendly process. These treatments modify surface energy or alter chemical bonds, enabling the deposition of thin films with excellent adhesion and substrate compatibility.^19,26–28^ Plasma-Enhanced Chemical Vapor Deposition (PECVD) stands out as a clean, dry, and highly controllable process.^29^ The use of organosilanes, such as hexamethyldisiloxane (HMDSO), has been widely investigated for thin film deposition across various fields, as it enables the formation of hybrid layers containing siloxane groups (−Si–O−)—which are compatible with glass and metals—and functional organic groups capable of interacting with the Polymer.^30–33^ Furthermore, oxygen plasma post-treatments can functionalize these surfaces, enhancing wettability and chemical reactivity.^34–37^

While the use of HMDSO is well-established for corrosion protection and electronic applications, its specific application for optimizing metal–polymer interfaces in welding remains underexplored, particularly regarding the monomer plasma polymerization parameters and their implementation in welded composite joints. Inside of this context, this study aims to enhance the shear strength of GF/PEI composite welded joints by depositing HMDSO thin films onto the stainless-steel mesh (AISI 304). A Taguchi L9 experimental design was employed to optimize plasma deposition parameters (power, time, and composition), followed by an investigation into the effect of an O_2_ plasma post-treatment. Treatment efficacy was evaluated via physicochemical characterization (FTIR, XPS, and Contact Angle) and Single Lap Shear Strength (SLSS) tests, correlating the resulting surface properties with fracture morphology and welded joint performance.

## 2 Methodology

### 2.1 Materials and Manufacturing

The laminate composite used in this study was Glass Fiber-Reinforced Poly­(ether imide) (GF/PEI), supplied by Toray Advanced Composites. The material features a (0°/90°)_5_s lay-up configuration with an 8HS fabric weave, a matrix volume fraction of 50%, and a nominal thickness of 2.0 mm. An AISI 304 stainless-steel mesh was employed as the Heating Element (HE), characterized by 400 openings per inch, a wire diameter (*d_x_*) of 25 μm, an aperture size (φ*_x_*) of 39 μm, and an open area fraction (*F*_0_) of 37%. As illustrated in Figure 1, the PEI molecular structure is presented.

**Figure 1:**
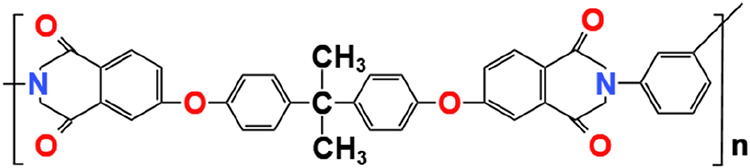
Monomer of polyethylene (C_37_H_24_O_6_N_2_)­n.

### 2.2 Surface Modification

For the HE surface modification, hexamethyldisiloxane (HMDSO) was selected as the precursor monomer for thin film deposition via plasma. HMDSO is an organosilane widely employed in plasma-assisted deposition processes due to its ability to form hybrid layers containing siloxane groups (−Si–O−) and organic functionalities, imparting properties such as hydrophobicity, chemical resistance, and enhanced adhesion. The HMDSO thin films were deposited onto the HE via plasma-enhanced chemical vacuum deposition (PECVD) in an asymmetric plasma reactor (Figure 2).

**Figure 2:**
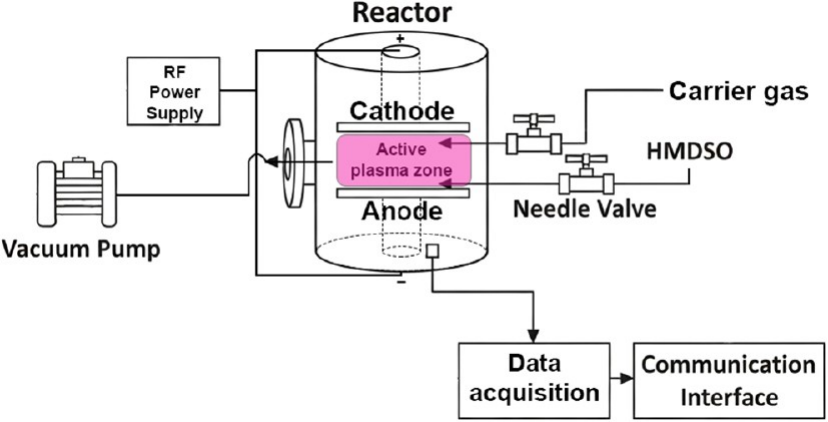
Schematic representation of the low-pressure plasma reactor.

For the deposition process, the HE was subjected to five steps: (I) Cleaning in an ultrasonic bath with methanol for 30 min to remove oils and grease. Subsequently, the HE was dried in an oven at 80 °C for 15 min. (II) The HE was positioned parallel to the gas flow direction, and the chamber was evacuated until the base pressure reached 1.6 × 10^–2^ Torr. (III) Argon was introduced into the chamber with a controlled flow until the internal pressure stabilized at 10^–1^ Torr. Next, (IV) an electric power of 30 W was applied by using a radiofrequency (RF) power supply at a standard frequency of 13.56 MHz. The argon plasma was maintained for 10 min, assisting in the removal of surface impurities from both the substrate and the chamber walls. This process also increased the surface energy of the substrate, enhancing its wettability. After this period, the plasma was extinguished, and the system was re-evacuated. Finally, (V) HMDSO was introduced in the vapor form with a controlled flow. The mixture was balanced with argon until the reactor’s internal pressure stabilized at 10^–1^ Torr. Several combinations of gas ratio, power, and exposure time were evaluated to identify the optimal parameters based on previous studies.^38–40^ Table 1 presents the Taguchi L9 experimental design adopted for the classification and optimization of these variables.

**Table 1:** Taguchi Planning L9 for HMDSO Deposition

**matrix of variables and levels**						
	**coded**			**real**		
**standard**	**times** (min)	**composition** (%)	**power** (W)	**time** (min)	**composition** (%)	**power** (W)
1	–1	–1	–1	20	20	10
2	–1	–1	0	40	20	30
3	–1	0	1	60	20	50
4	0	–1	0	40	60	10
5	0	0	1	60	60	30
6	0	1	–1	20	60	50
7	1	–1	1	60	80	10
8	1	0	–1	20	80	30
9	1	1	0	40	80	50

A second sample group, based on the optimal condition derived from the orthogonal array, was subjected to an O_2_ plasma post-treatment using a gas mixture of 80% Argon and 20% Oxygen. The Argon/Oxygen ratio was maintained at a fixed 80/20% for a working pressure of 1 × 10^–1^ Torr. The selection of these concentrations for the O_2_ plasma post-treatment was based on previous studies.^34,36,37,41–43^

### 2.3 Film Characterization

The films were characterized considering two specific conditions: (i) as-deposited HMDSO and (ii) HMDSO followed by O_2_ plasma post-treatment. To ensure inert substrates, both batches were deposited onto aluminum foil for FTIR-ATR analysis and onto polished silicon wafers for AFM and ellipsometry analyses.

Chemical composition and functional groups were investigated by Fourier Transform Infrared Spectroscopy with Attenuated Total Reflectance (FTIR-ATR), using a PerkinElmer Spectrum 100 spectrometer. Spectra were acquired in the range of 4000 to 650 cm^–1^, with a resolution of 4 cm^–1^ and 12 scans to optimize the signal-to-noise ratio. Elemental analysis and surface chemical state were determined by X-ray Photoelectron Spectroscopy (XPS), employing a Thermo Scientific K-α system.

Atomic Force Microscopy (AFM), performed on a Shimadzu SPM 9600 instrument, was used to evaluate the film topography via phase imaging. For each condition, five random 5 × 5 μm regions were analyzed, and data were processed using Gwyddion software. Film thickness was measured by ellipsometry using a Horiba Uvisel II spectroscopic ellipsometer.

Wettability properties were assessed by static contact angle measurements using a Ramé-Hart 300-F1 goniometer with 1 μL drops of deionized water. Angles lower than 90° indicate hydrophilic character, while angles greater than 90° denote hydrophobic behavior. Surface aging was monitored through sequential contact angle measurements over time.

### 2.4 Welding Procedure

The Single Lap Shear Strength (SLSS) specimens were cut to dimensions of 100 × 25.4 mm (Figure 3a). The welding area was defined as 25.4 × 25.4 mm (Figure 3b), in accordance with ASTM D1002–10 and ASTM D5868–01 standards. Prior to welding, the composites were cleaned with isopropyl alcohol and conditioned in an oven at 80 °C for 12 h to remove absorbed moisture.

**Figure 3:**
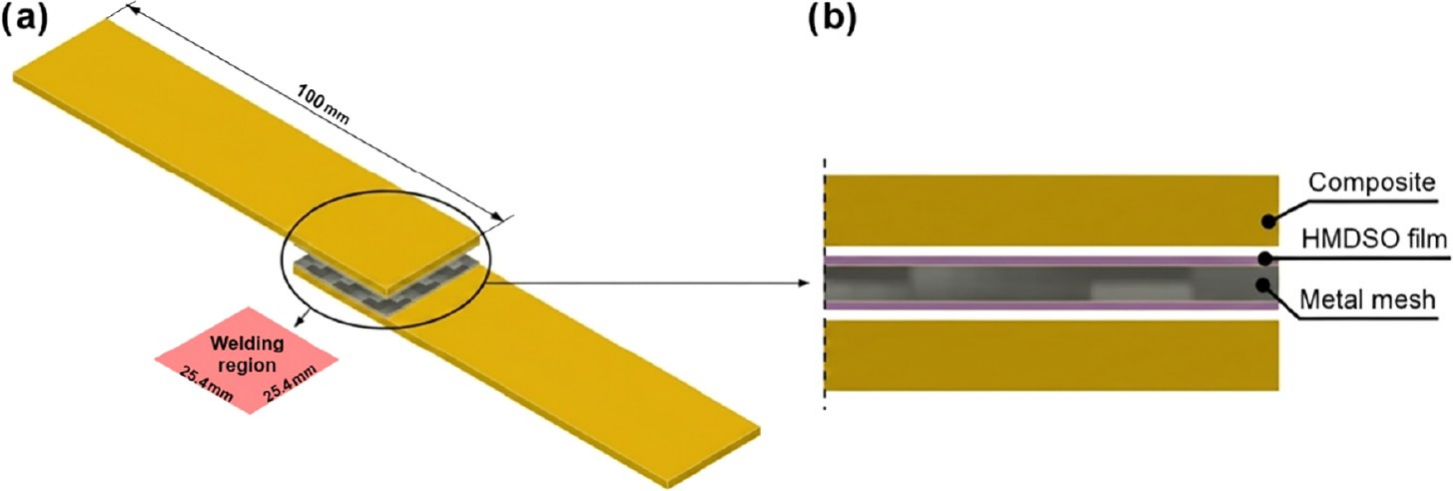
Representation of the sample for SLSS testing: general geometry (a); the sandwich structure has been magnified to show its components in greater detail (b).

The resistance welding parameters were selected based on previous studies with GF/PEI:^44^ a current of 25 A, a pressure of 3.7 MPa, a power density of 1600 kW/m^2^, and a welding time of 35 s, followed by 60 s under pressure after current termination.

### 2.5 Characterization of Welded Joints

Single Lap Shear Strength (SLSS) tests were conducted on a Shimadzu AGX-50kN Universal Testing Machine (UTM), equipped with a 50 kN load cell, at a crosshead speed of 1.5 mm/min until joint failure.

To analyze the welded interface, as-welded specimens were sectioned longitudinally and observed via reflected light optical microscopy. Following mechanical testing, the fracture surfaces were examined by using the same method. Low-magnification analysis was performed on a Zeiss Stemi 2000 stereomicroscope, while higher magnifications and images with extended depth of focus reconstruction were obtained by using a Zeiss Axio Imager Z2m optical microscope.

Detailed fractographic characterization was carried out via Scanning Electron Microscopy (SEM) using a Zeiss EVO LS15, acquiring both secondary and backscattered electron signals to identify morphological features and failure mechanisms. To preserve the original fracture morphology and contrast and also to avoid potential masking of fine surface details by a conductive coating, the fractured composite surface was analyzed under the variable pressure (or low vacuum) mode. This mode enables charge neutralization in nonconductive materials without the need for metal deposition, ensuring the collection of a clear topographic signal directly from the sample surface.

## 3 Results and Discussion

### 3.1 Taguchi Experimental Design Analysis

Table 2 presents the experimental results obtained from the combination of deposition variables. The effects of these parameter combinations, similar to those investigated for HMDSN thin film deposition via PIIID by,^39^ showed trends comparable to those observed in the current study.

**Table 2:** Experimental Response of the LSS Test for Deposition Combinations

**Taguchi L9 matrix response for HMDSO deposition**							
	**codificado**			**real**			**response**
**standard**	**time** (min)	**composition** (%)	**power** (W)	**time** (min)	**composition** (%)	**power** (W)	**LSS** (MPa)
1	–1	–1	–1	20	20	10	13.75 ± 0.66
2	–1	–1	0	40	20	30	13.00 ± 0.84
3	–1	0	1	60	20	50	8.79 ± 0.14
4	0	–1	0	40	60	10	7.80 ± 0.64
**5**	**0**	**0**	**1**	**60**	**60**	**30**	**14.58 ± 0.29**
6	0	1	–1	20	60	50	9.60 ± 2.21
7	1	–1	1	60	80	10	10.39 ± 0.39
8	1	0	–1	20	80	30	13.36 ± 0.39
9	1	1	0	40	80	50	5.40 ± 1.61

A wide variation in Lap Shear Strength (LSS) values was observed, ranging from 5.41 to 14.58 MPa, indicating that deposition parameters substantially influence the mechanical performance of the specimens. The highest LSS value was achieved in Experiment 5, corresponding to the intermediate condition for all factors (60 min, 60% HMDSO, and 30 W), as highlighted in red in Table 2. A normality analysis of the LSS response was performed to verify whether the experimental data follow an approximately normal distribution and to confirm that the observed variability is not attributable to experimental error. The near-linear alignment of the data points with the theoretical normal dispositions indicates that the LSS response is normally distributed, as can be seen in Figure 4. Therefore, despite the wide variation observed in the measured values, the normal probability plot supports the absence of significant asymmetry or extreme outliers in the LSS data and validates the application of parametric statistical methods as variance and signal-to-noise analysis.

**Figure 4:**
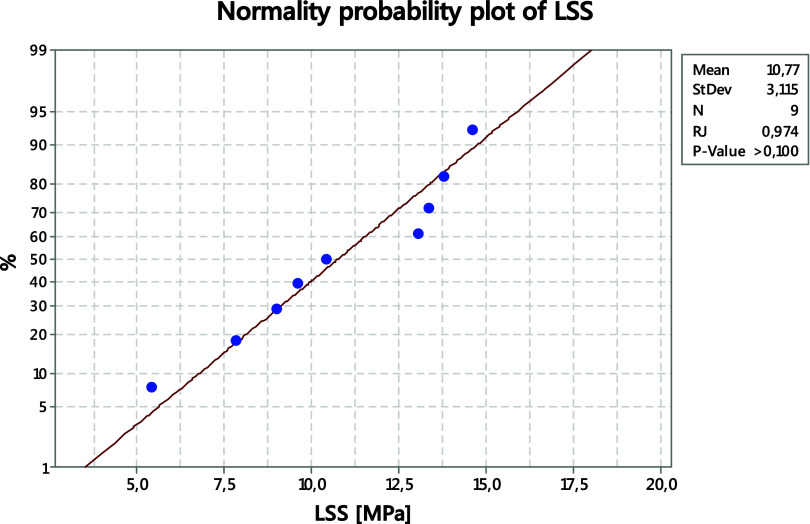
Normality probability plot of LSS results.

Upon analyzing the mean effect trends for each factor (Figure 5), it was observed that HMDSO composition exhibits a decreasing trend: higher precursor levels (80%) resulted in lower LSS. This behavior suggests that excessive HMDSO concentrations may yield films with lower cross-linking density or higher organic content,^45,46^ thereby impairing adhesion.^46,47^ Consequently, intermediate compositions (60%) proved to be more favorable for mechanical performance.

**Figure 5:**
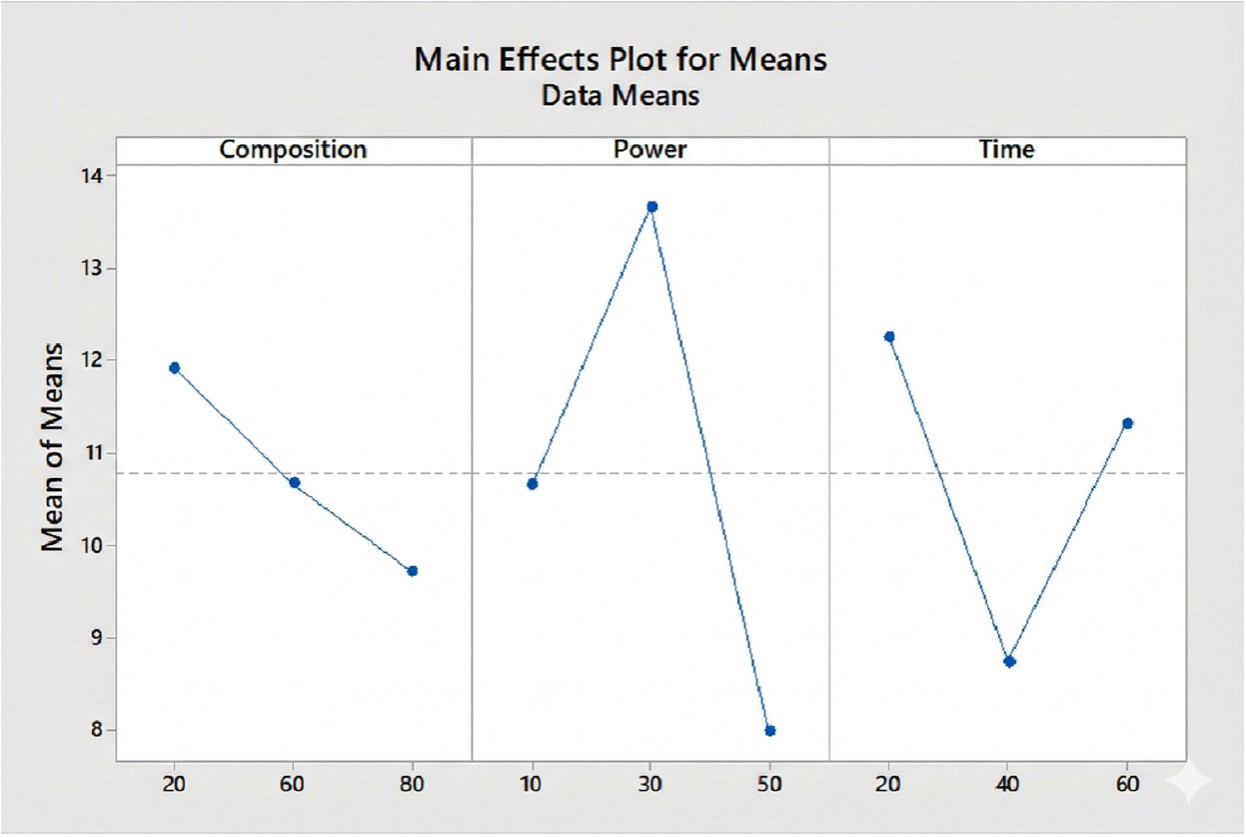
Influence of HMDSO polymerization variables.

The analysis of main effects and variance (ANOVA) of the *S*/*N* ratio values (Table 3) quantifies this influence, identifying applied power as the most significant factor (*p* = 0.020), followed by deposition time (*p* = 0.042). HMDSO composition exhibited a marginal effect (*p* = 0.083). Power demonstrated the primary influence on the response with the intermediate level of 30 W yielding the highest mean SLSS, whereas high power (50 W) led to a significant decrease. This behavior is consistent with the literature, which suggests that excessive power levels can degrade the surface or generate more brittle films due to excessive ion energy, thereby compromising adhesion.^29^

**Table 3:** Analysis of Variance for SN Ratios

**factor**	**DF**	**seq SS**	**adj SS**	**adj MS**	***F*-value**	***P*-value**
composition	2	12.103	12.103	6.0513	11.05	0.083
power	2	52.848	52.848	26.4239	48.25	0.020
time	2	25.284	25.284	12.6419	23.08	0.042
error	2	1.095	1.095	0.5477		
total	8	91.330				

The response table for the signal-to-noise ratios (Table 4), considering the statistical criterion of ″larger is better,” indicates that power is the factor with the greatest influence on the Lap Shear Strength (LSS) values. This result demonstrates that controlling the power parameter is the most effective approach to ensure result reliability and improve process stability, as it exhibits the highest impact on the combination of deposition variables.

**Table 4:** Response Table for Signal-to Noise Ratios (Larger is Better)

**level**	**composition**	**power**	**time**
1	21.31	20.21	21.23
2	19.80	22.63	17.50
3	18.47	16.73	20.85
delta	2.84	5.90	3.73
**rank of factor impact**	**3**	**1**	**2**

Regarding precursor composition, a clear decreasing trend was observed, where the highest HMDSO concentration (80%) systematically resulted in the poorest performance. This suggests that an excess of precursor favors the formation of films with a more polymeric and less cross-linked character, reducing the adhesion capacity and rendering intermediate concentrations (∼60%) more favorable.

The effect of deposition time exhibited a nonlinear relationship. Although the intermediate time of 40 min showed the lowest response on average, the longer time of 60 min, when combined with optimal composition and power levels (Exp. 5), resulted in the best performance. Experimental observations helped elucidate this behavior: for times exceeding 30 min, particle formation was noted in the reactor and on the specimens, indicating growth saturation and film fragmentation. A validation experiment, reducing the time of Exp. Five to 30 min, confirmed this hypothesis, maintaining values similar to the maximum mean SLSS (14.58 MPa). This result demonstrates that shorter deposition times (30 min), provided they are associated with ideal composition and power, are sufficient to produce high-quality films, avoiding the supersaturation and discontinuity observed in prolonged depositions.

The thickness of the HMDSO films deposited by PECVD was determined by profilometry, as presented in Figure 6. The O_2_ plasma post-treatment resulted in a thickness reduction from approximately 502 nm (untreated film) to 475 nm, representing a decrease of 27 nm (5.2%).

**Figure 6:**
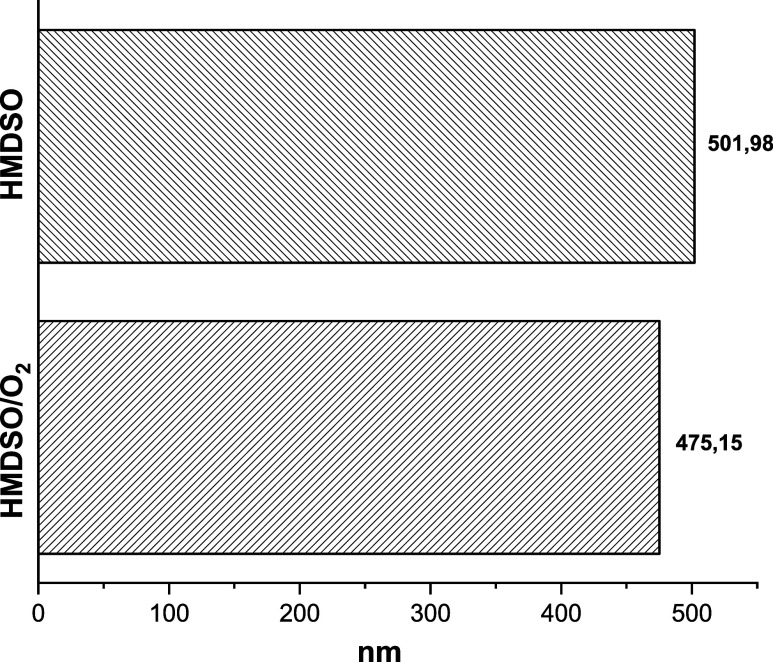
Film thickness in different configurations.

This behavior is attributed to simultaneous physical and chemical mechanisms. Energetic ion bombardment promotes surface ablation via sputtering, preferentially removing carbon-rich regions. Concurrently, oxidation reactions replace bulky methyl groups (−CH_3_) with siloxane bridges (Si–O–Si) and hydroxyl groups, leading to network compaction and the elimination of volatiles (CO_2_ and H_2_O). This structural densification, combined with the transition toward a more inorganic film character (SiO*_x_*), aligns with the dimensional stability and wettability modifications previously observed, occurring without excessive mechanical degradation of the deposited layer.

### 3.2 Coating

#### 3.2.1 Chemical Characterization

The chemical characterization via FTIR (Figure 7) confirmed the organosiloxane structure of the deposited film, evidenced by intense bands between 1250 and 850 cm^–1^ (Si–O–Si and Si­(CH_3_)_2_ stretching vibrations) and at 2900 cm^–1^ (C–H stretching of methyl groups). The O_2_ plasma treatment induced significant spectral modifications: the emergence of bands associated with silanol groups (Si–OH) was observed at 3200–3500 cm^–1^, alongside a relative increase in the intensity of inorganic bands (Si–O–Si) at the expense of organic peaks (−CH_3_).^48^

**Figure 7:**
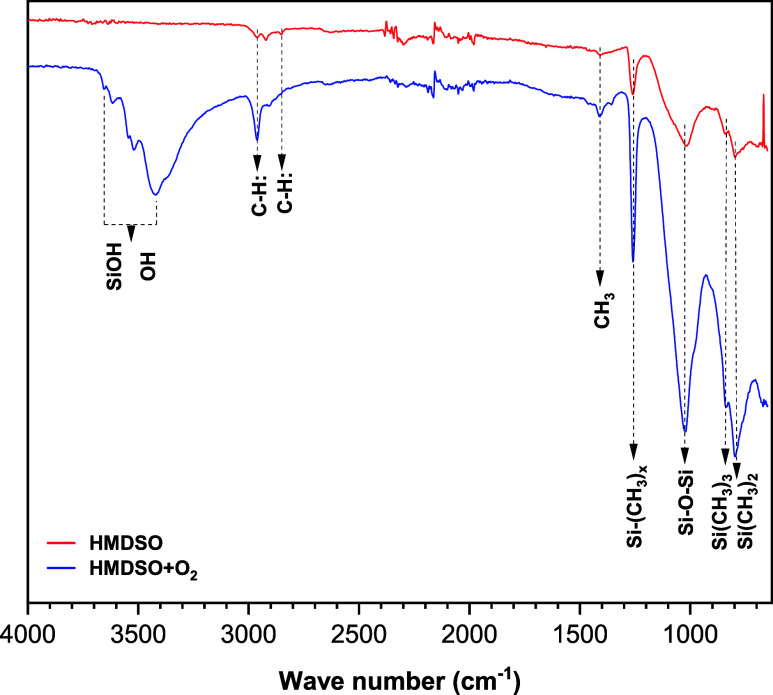
Functional groups detected by FTIR analysis.

The analytical data support a mechanism based on surface modification rather than sequential deposition. Upon exposure to an O_2_ plasma, reactive oxygen species (radicals and ions) bombard the previously deposited organosiloxane film. Consequently, hydrocarbon moieties (CH_3_) are progressively abstracted and volatilized as CO and CO_2_.^31^ The resulting vacancies are subsequently occupied by oxygen atoms, promoting the formation of new Si–O–Si bonds (cross-linking). This process leads to the formation of a distinct bilayer structure: the underlying, ductile SiOxCyHz core remains polymeric, while the surface is effectively converted into a densified, hard, and silica-rich (SiO*_x_*) top layer.^49^ Thus, the treatment induces a vertical compositional gradient, shifting the surface chemistry toward a strictly inorganic character. However, given that FTIR is mainly qualitative—identifying bonds by peak position with intensity serving only as an indicator—rigorous quantitative analysis using XPS was necessary to assess the differences in elemental ratios.

XPS analysis (Figure 8) revealed a drastic transformation in the surface composition. The atomic fraction of carbon decreased significantly (from ∼35.30 to ∼11.50%), confirming the efficient removal of methyl groups. Consequently, driven by this selective mass removal and the incorporation of oxygen-functionalized groups, the relative atomic fraction of oxygen increased to ∼48%, while the silicon content remained stable.

**Figure 8:**
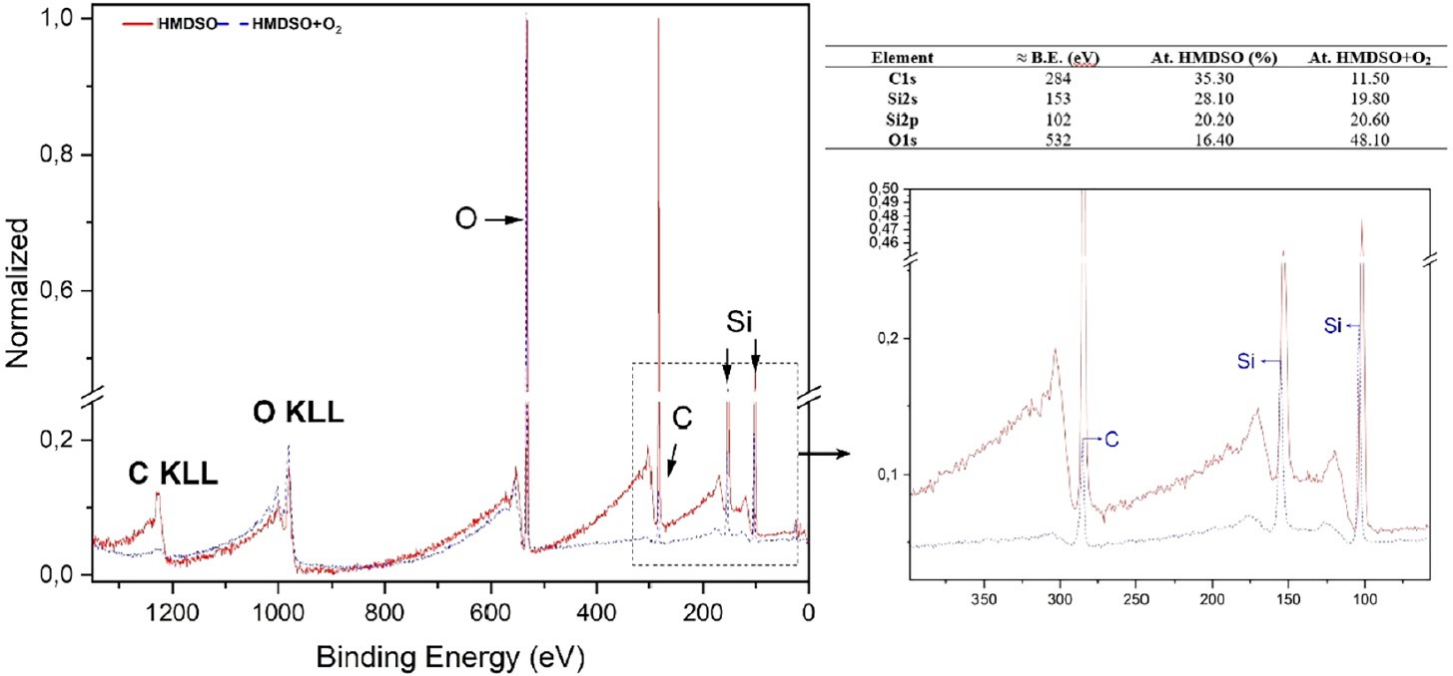
XPS survey spectra and elemental composition of the HMDSO films before and after O_2_ plasma treatment. The inset highlights the variations in C 1s and Si peaks in the 50–400 eV range. The table summarizes the atomic concentration percentages and approximate binding energies.

Furthermore, high-resolution analysis of the Si 2p peak (Figure 9) showed a binding energy shift and an increase in the relative intensity of the component associated with Si­(O)_4_, at the expense of lower oxidation states such as Si­(O)_1_. This indicates a deeper oxidation of the silicon network, converting Si–CH_3_ bonds into oxygen-rich structures and shifting the film composition toward a SiO_2_-like structure, consistent with literature observations.^35,50^

**Figure 9:**
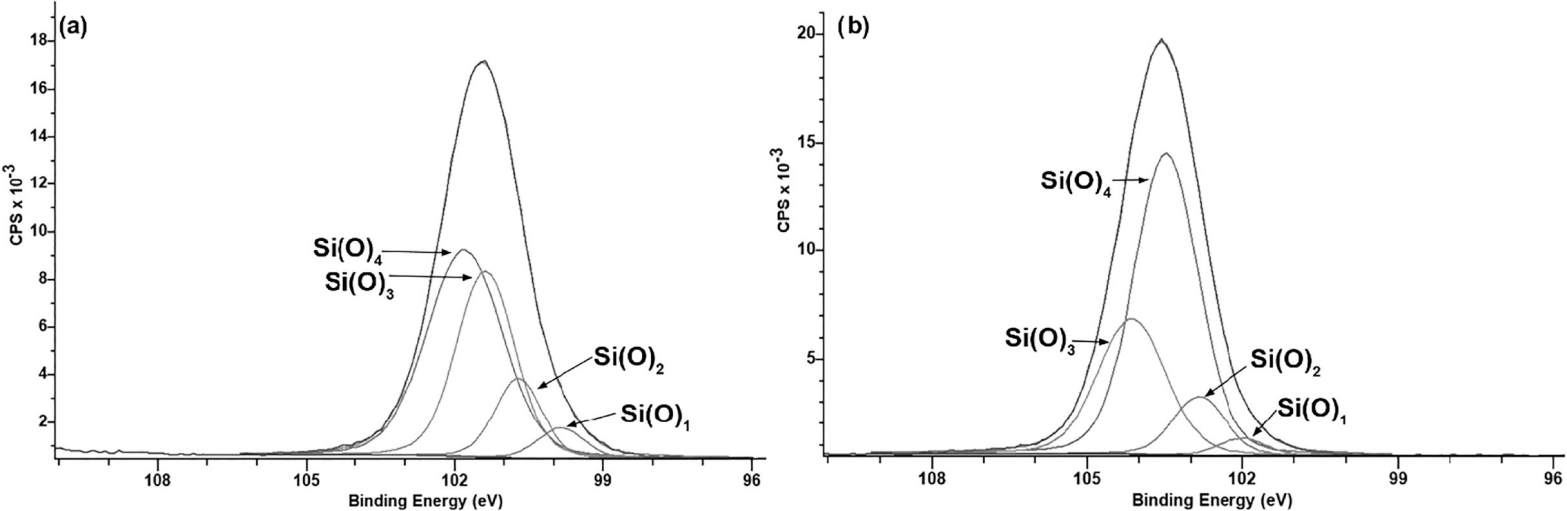
XPS high-resolution analysis of Si peak of HMDSO (a) and HMDSO+O_2_ (b).

This effect is further supported by the high-resolution O 1s analysis (Figure 10), where peaks shifted from binding energies corresponding to C=O and C–O bonds to those related to Si–O–Si and SiO_*x*_ ^51–54^

**Figure 10:**
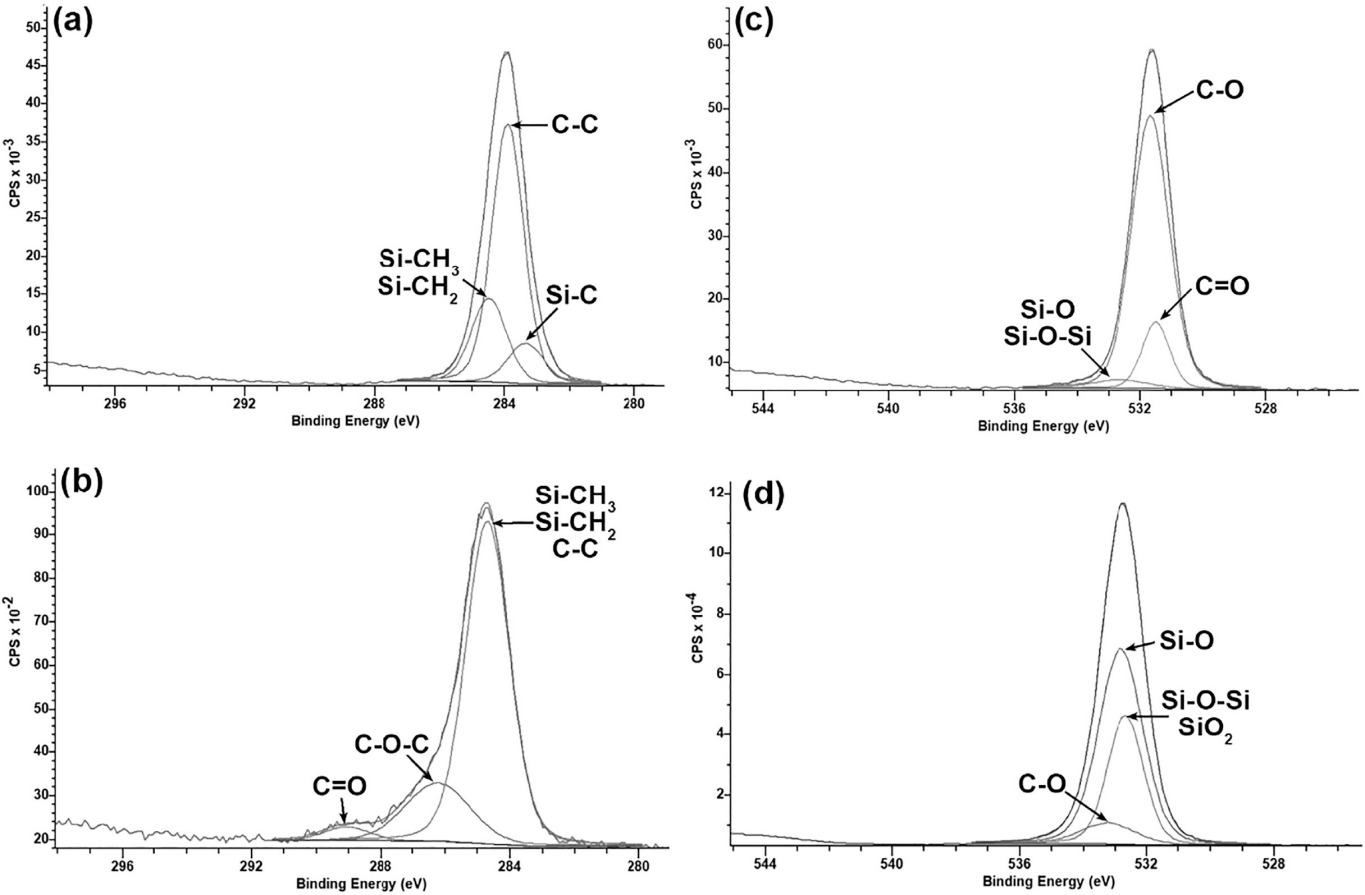
High-resolution XPS spectra comparing C 1s and O 1s regions before and after plasma treatment. (a, b) C 1s peaks for pristine HMDSO and HMDSO+O_2_, respectively. (c, d) O 1s peaks for pristine HMDSO and HMDSO+O_2_, respectively.

A proposed reaction mechanism for film formation and subsequent surface functionalization is presented, as illustrated in Figure 11. This proposal is based on the dissociation patterns that were observed in the FTIR and XPS analyses. During the deposition phase, the plasma energy fragments the HMDSO monomer, leading to the formation of a cross-linked polysiloxane network containing hydrophobic methyl groups. The subsequent O_2_ plasma treatment promotes the oxidation of this surface, preferentially removing organic moieties (−CH_3_) and creating a silica-like layer rich in polar active sites (such as Si–OH and Si–O–C), which are critical for enhancing the chemical affinity with the PEI matrix.^36,39,49,55^

**Figure 11:**
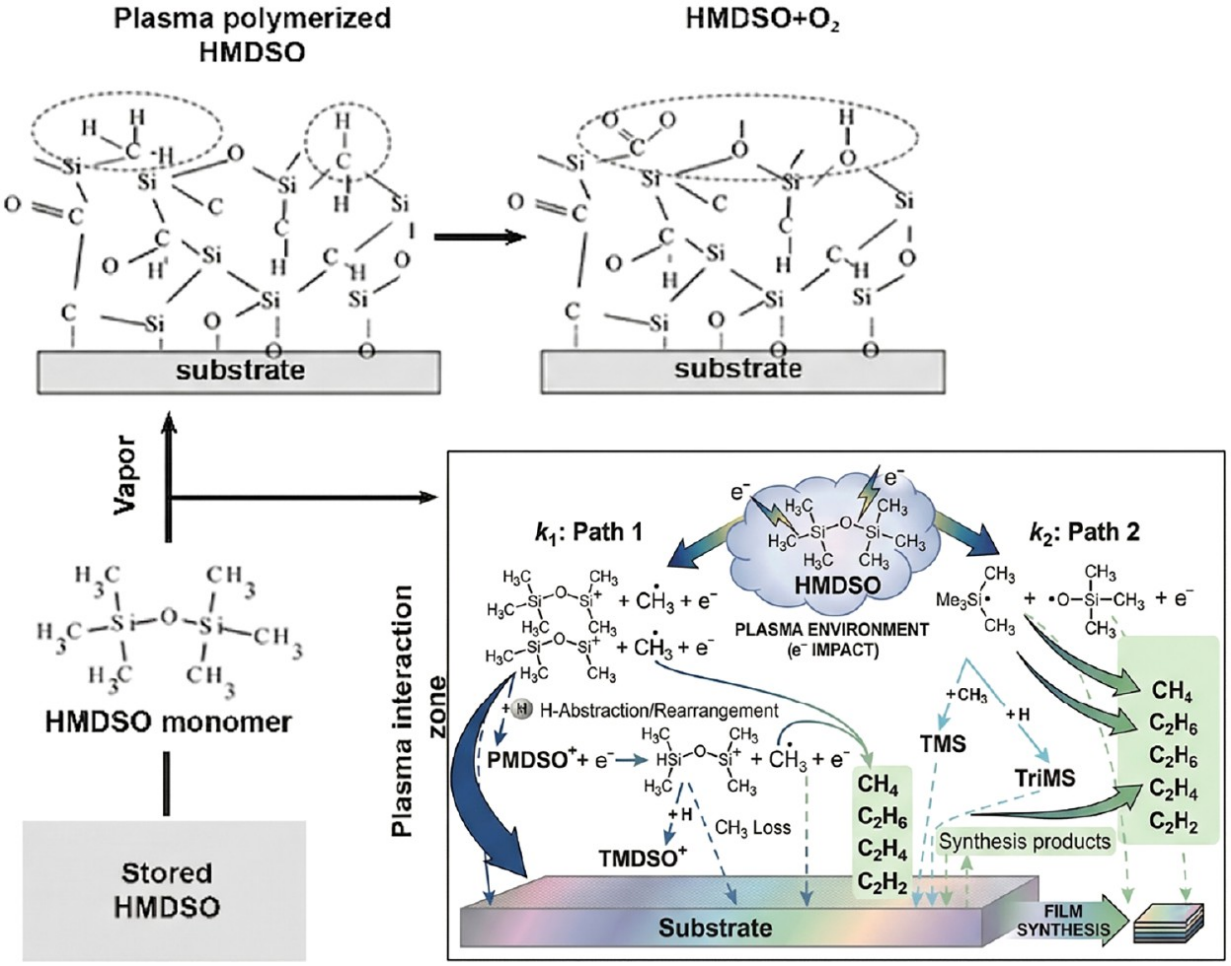
Schematic representation of HMDSO dissociation pathways during PECVD and the resulting surface molecular structure before and after oxygen plasma treatment. Adapted with permission from Yoshinari, M.; Matsuzaka, K.; and Inoue, T. Surface modification by cold-plasma technique for dental implants—biofunctionalization with binding pharmaceuticals. *Jpn. Dent. Sci. Rev.* 2011, **47**, 89–101. Copyright 2011 Japanese Association for Dental Science CC BY 4.0 license.

The direct impact of these chemical changes on wettability was clearly demonstrated by contact angle measurements (Figure 12). The incorporation of oxygen-containing groups and the conversion of CH_3_ groups, as evidenced by FTIR and XPS analyses, decisively modified the film’s wettability properties, rendering it more hydrophilic immediately after treatment. This increased hydrophilicity makes the surface more suitable for applications requiring higher affinity with polar environments and improved chemical adhesion,^50,56^ being particularly advantageous for joining with polymeric matrices.

**Figure 12:**
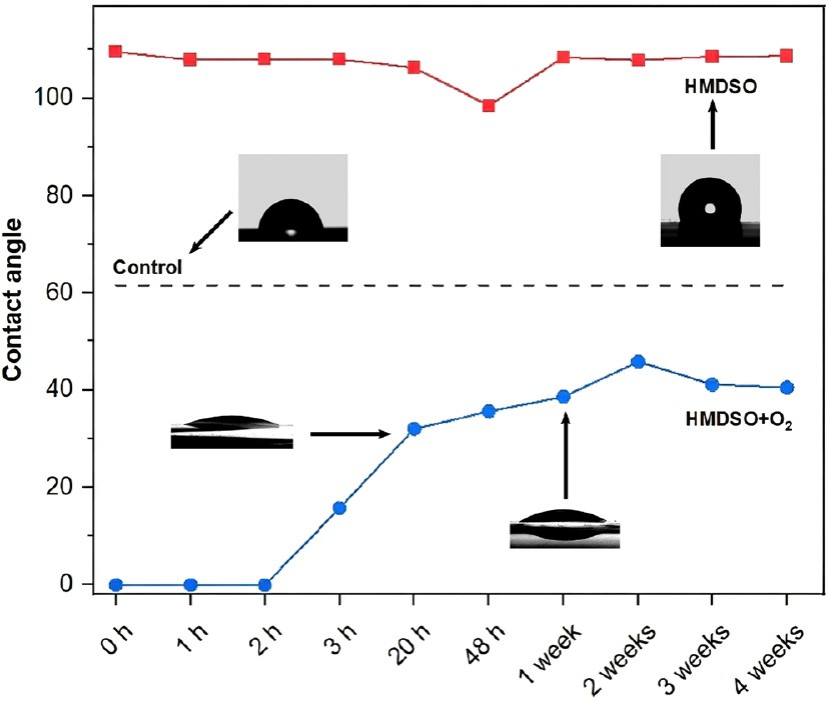
Contact angle evolution over time.

As expected, the as-deposited HMDSO film exhibited a stable hydrophobic behavior over time. In contrast, the film subjected to O_2_ plasma post-treatment displayed the aforementioned hydrophilic character, followed by a process of hydrophobic recovery. The contact angle increased over time until stabilizing after approximately 168 h at a value of about 40°. Although higher than that observed immediately after treatment, this value remains significantly lower than that of the untreated film (61.6°) and the control, imparting lasting hydrophilicity. This recovery is attributed to the dynamic reorganization of surface polymeric chains and the potential slow oxidation of the introduced functional groups—processes that gradually reduce surface energy and its polar component. However, the permanent oxidation of the network ensures that the final wettability remains superior to that of the initial condition.

#### 3.2.2 Profilometry

The surface topography of the films was evaluated via Atomic Force Microscopy (AFM).

Table 5 summarizes the roughness parameters Sa (arithmetic mean height) and Sq (root-mean-square height) observed in the analyzed regions. The high standard deviation and the marked discrepancy between Sa and Sq indicate a heterogeneous surface with a nonuniform height distribution. This behavior is likely due to gas-phase particulate formation, a phenomenon typical of organosilicone PECVD processes under certain power regimes.^57^ The films exhibit morphological features characteristic of HMDSO thin films, effectively preserving the substrate characteristics with roughness largely dependent on the substrate to which they were applied.

**Table 5:** Mean Sa and Sq Values of the Areas Scanned by AFM

	**Sa (nm)**	**Sq (nm)**
**HMDSO**	2.98 ± 2.79	7.64 ± 9.39
**HMDSO+O**_**2**_	0.73 ± 0.09	1.54 ± 0.15

Figure 13 presents the 3D surface reconstructions. Morphological patterns can be influenced by parameters such as the type of plasma reactor used, gas nature, substrate exposure time, power, flow rate, and the type of source employed.^30,35,58^ Higher precursor and carrier gas flow rates as well as high frequencies may result in increased masking of the substrate surface, generating morphologies with more peaks and valleys yet with little variation in Sq values. This behavior was observed by Chaiwong,^35^ who reported Sq values between 0.66 nm (at 100 Hz) and 2.12 nm (at 400 Hz), attributed to the deposition of long, branched polymer chains. However, at 500 Hz, a reduction in Rq to 0.57 nm was observed due to high plasma energy that fragmented monomeric molecules and high-molecular-weight compounds, promoting a smoother surface.

**Figure 13:**
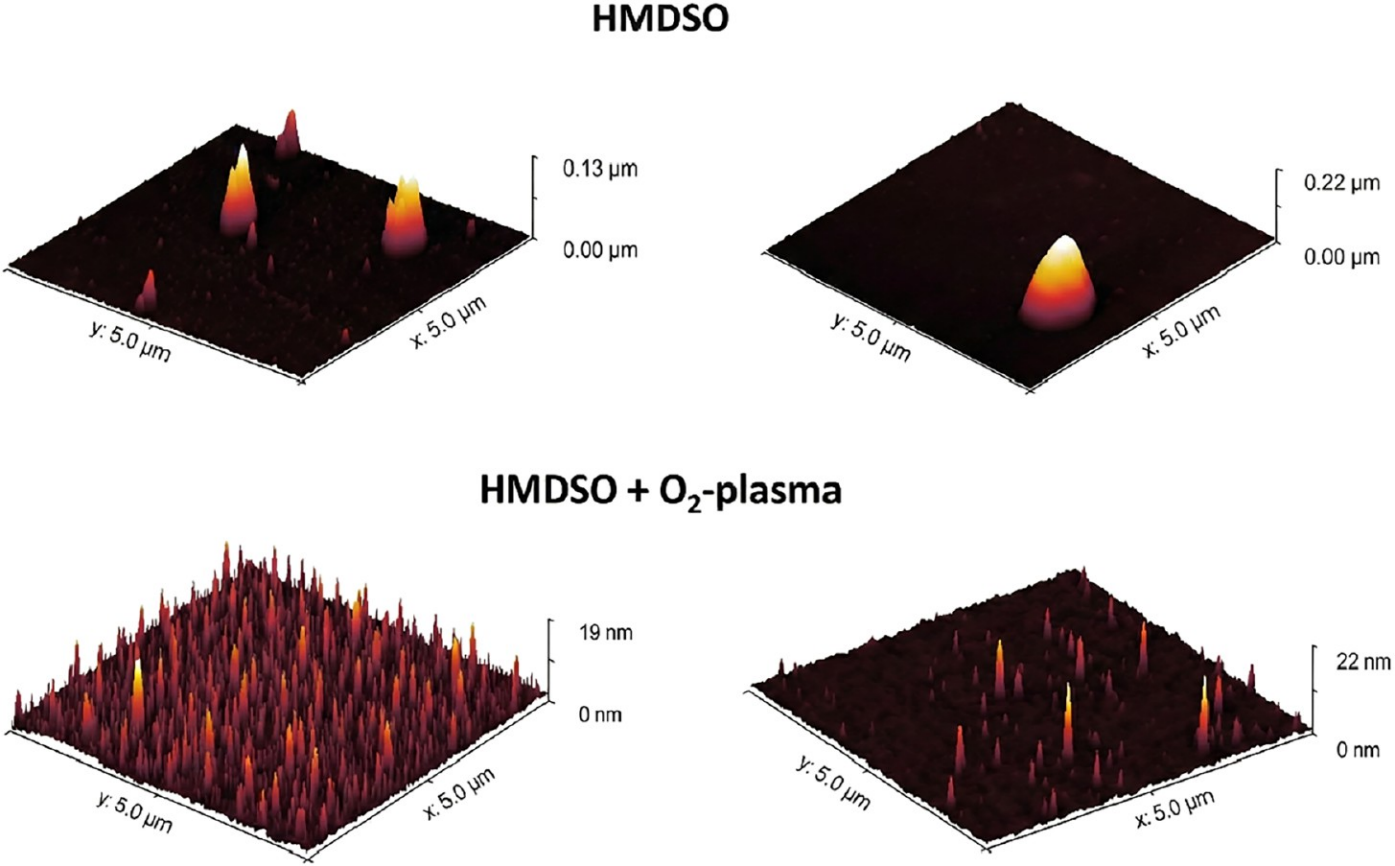
AFM images of HMDSO surfaces under different conditions.

The as-deposited HMDSO films exhibited an irregular morphology, characterized by the presence of agglomerates and high peaks. The plasma-deposited HMDSO layers reveal a granular or island-like growth morphology, characteristic of films with significant retention of organic groups (−CH_3_) and a relatively low oxidation level.^59,60^

After the O_2_ plasma treatment, the surface exhibits a visibly smoother and more uniform appearance. This smoothing effect is attributed to plasma action, occurring via two main processes: (i) physical ablation, where ion bombardment preferentially removes asperities and pendant organic groups (polishing effect); and (ii) chemical densification, where oxidation of methyl groups promotes the formation of a more compact and cross-linked inorganic SiOx network, consistent with XPS and FTIR results. Smoothing occurs because these peaks are more fragile, becoming preferential sites for bond breaking and molecule removal.

### 3.3 Welded Joint Characterization

Figure 14 presents the processing window of the primary process variables using the welding parameters adopted from the optimal Taguchi response. The temperature curve shows a progressive increase during the welding stage, surpassing the glass transition temperature Tg and reaching a peak of 221.6 °C within the programmed welding time. This behavior indicates that the composite was heated to a temperature sufficiently high to enable a polymer interface interaction.

**Figure 14:**
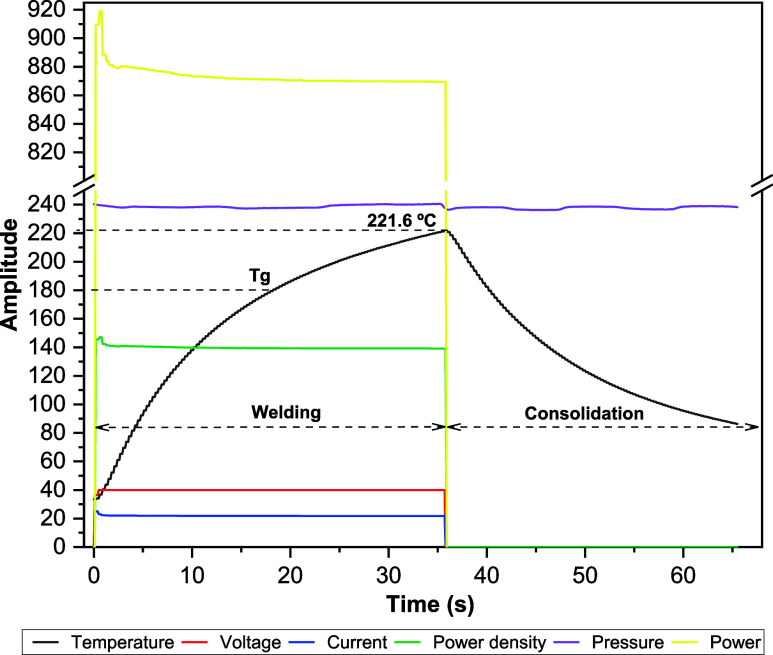
Processing window of the adopted welding parameters.

The welding stage, characterized by a temperature increase and stabilization of other variables, proceeds in a controlled manner, ensuring that the material reaches ideal conditions for coalescence without degradation. During this phase, current and voltage remain stable, guaranteeing a progressive energy input, as illustrated in Figure 15 via the integral of the electrical power curve over time, described by eq 1:

1 $$E=\int_{t1}^{t2}P\left(t\right)\mathrm{dt}$$

**Figure 15:**
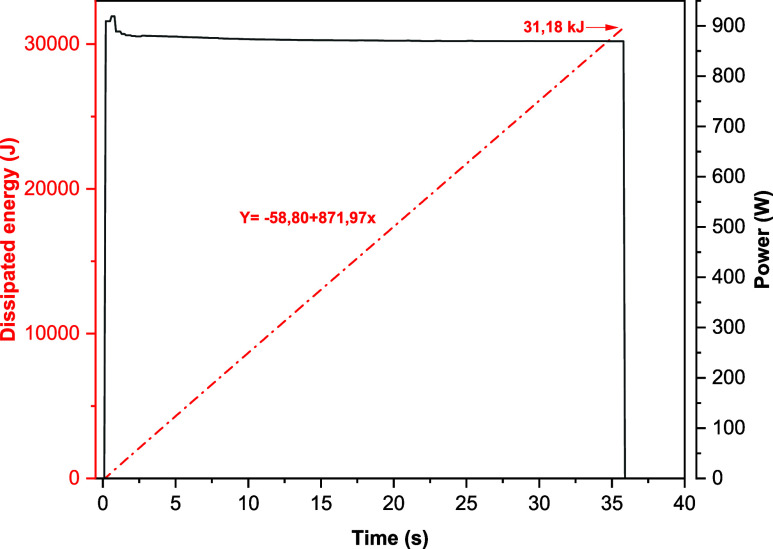
Energy dissipated in the welded joint.

In the consolidation phase, a gradual decrease in the temperature is observed, which favors the relief of internal stress and the solidification of the joined interface. The pressure applied throughout the cycle ensures intimate contact between the surfaces.^61^

The welded joints exhibited satisfactory consolidation features in their cross-section. The interfacial interaction regions displayed sound bonding characteristics, with the heating element fully embedded within the GF/PEI polymer matrix. A low incidence of voids or pores was observed across all welding conditions, limited to localized areas, as indicated by the red arrows in Figure 16. These observations confirm the effective consolidation of the joints and the thorough encapsulation of the stainless-steel heating element (bright metallic spots, green arrows) by the polymer matrix, for both untreated and HMDSO-treated samples.

**Figure 16:**
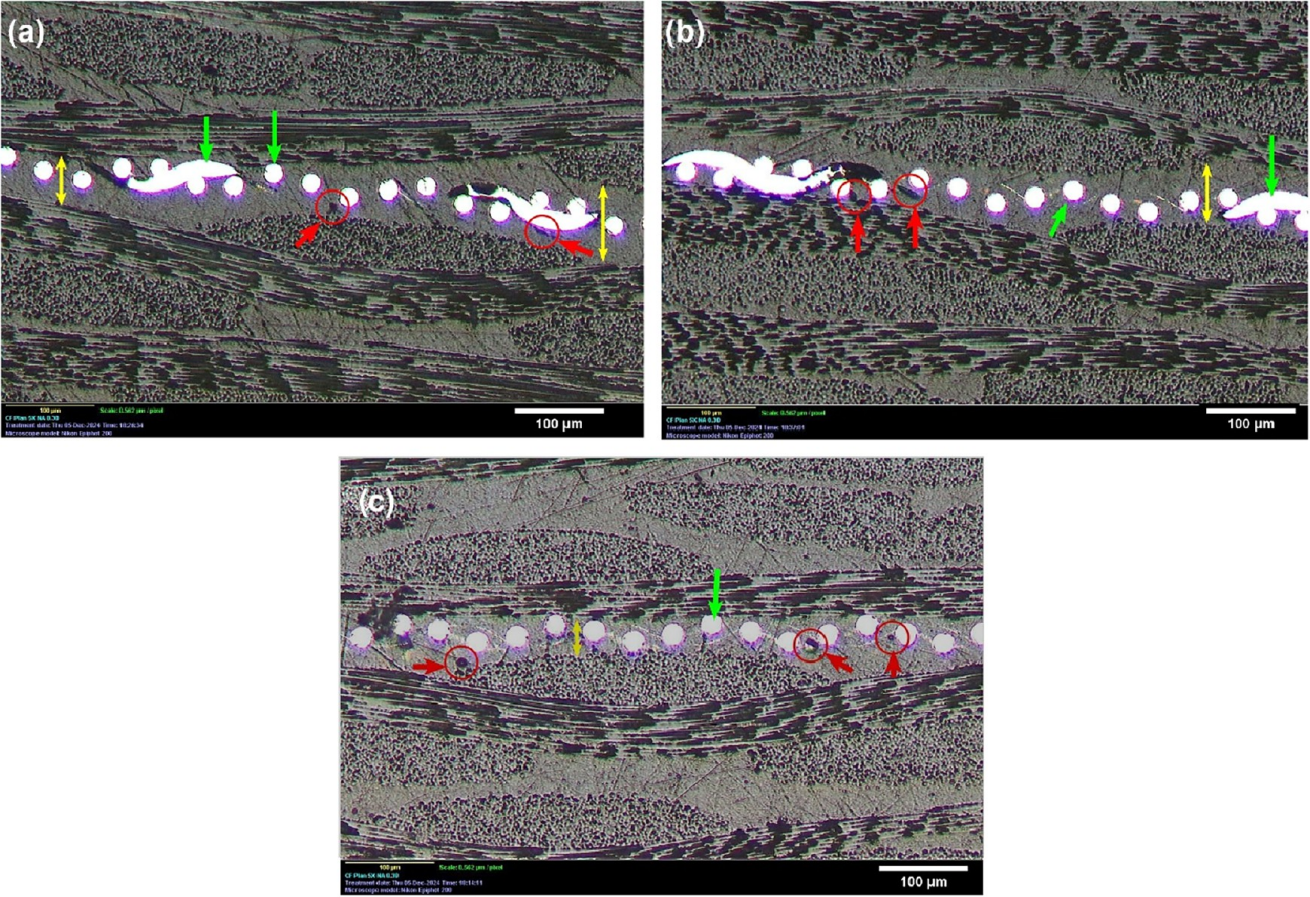
Longitudinal cross-section of welded joints: HMDSO (a), HMDSO+O_2_ (b), and untreated (c).

A higher concentration of such defects would indicate poor consolidation and could lead to joint embrittlement by acting as crack propagation sites.^6,14^ While this visual inspection does not comprehensively define the total mechanical quality of the joints—which requires fracture surface analysis following mechanical testing—it serves as a reliable indicator of bond integrity and interfacial interaction. The interface analysis focused on the resin-rich region surrounding the heating element (yellow arrows) to strictly distinguish actual voids from the natural interstices of the 0/90° fiber orientation.

### 3.4 Mechanical Behavior of the Joints

The application of HMDSO thin films and their subsequent post-treatment with an O_2_ plasma promoted a significant increase in the shear strength of the joints. As shown in Figure 17, the average SLSS values increased by 39.8% for the HMDSO film and by 48.0% for the HMDSO film with O_2_ post-treatment compared to the control sample.

**Figure 17:**
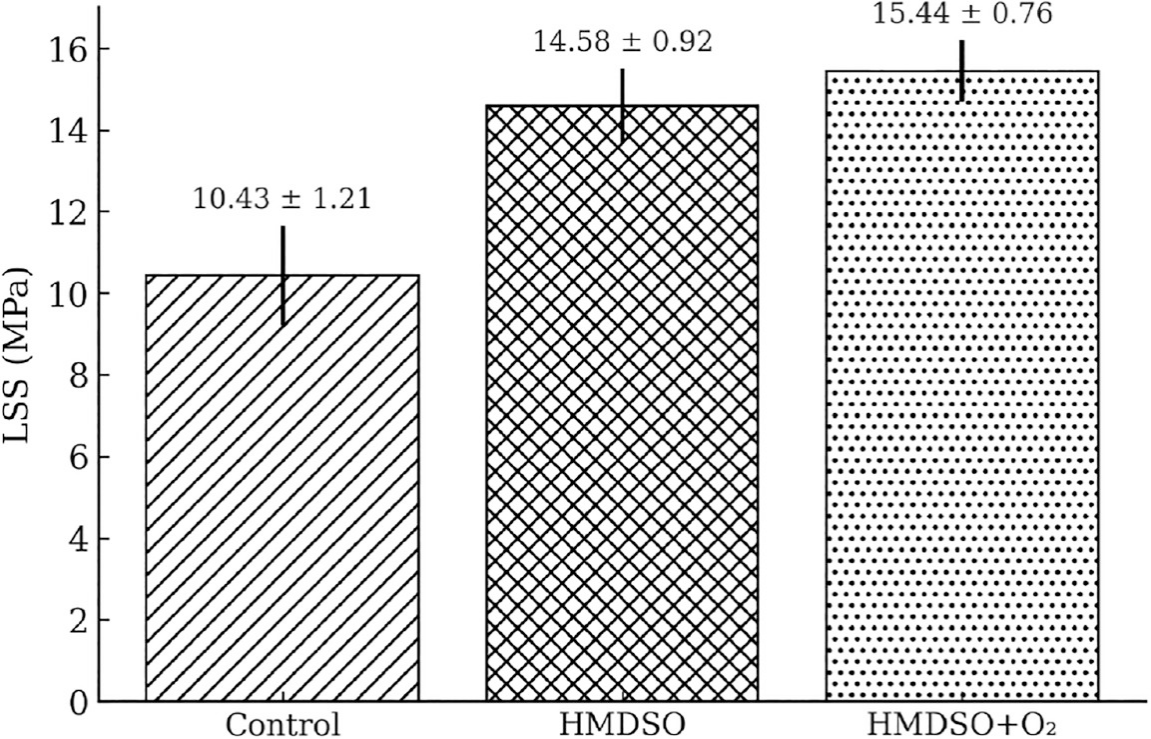
Average SLSS.

In addition to the strength increase, both surface treatments resulted in greater homogeneity of the welding process, evidenced by the remarkable reduction in the SLSS value dispersion (Figure 18). This reduced variability indicates superior control over interface quality and more reproducible adhesion.

**Figure 18:**
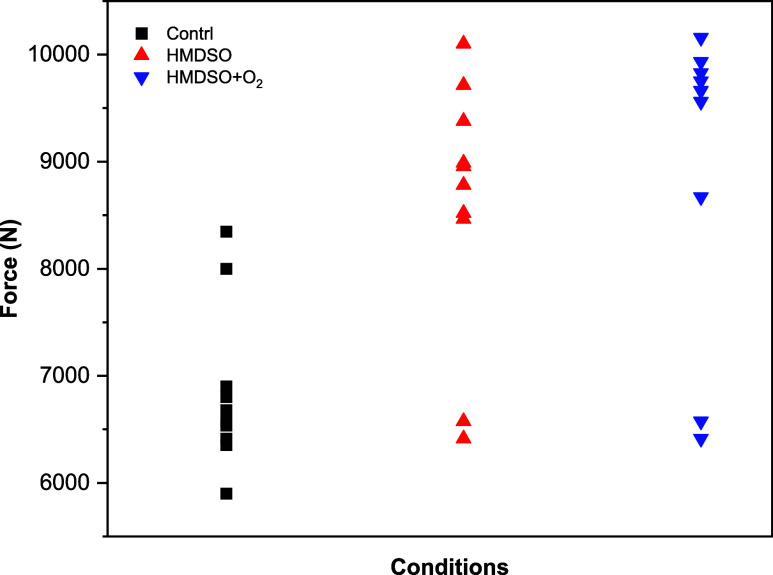
Scatter plot of individual SLSS values for the 10 samples of each batch.

### 3.5 Microscopic and Fractographic Characterization

Low-magnification analysis enabled a general characterization of the fracture in the welded region, while higher magnifications allowed for the identification of fractographic features present in the fractured joints. The fractured regions observed at low magnification indicate good interaction between the interfaces of the involved elements for welded polymer composites,^62^ as illustrated in Figure 19.

**Figure 19:**
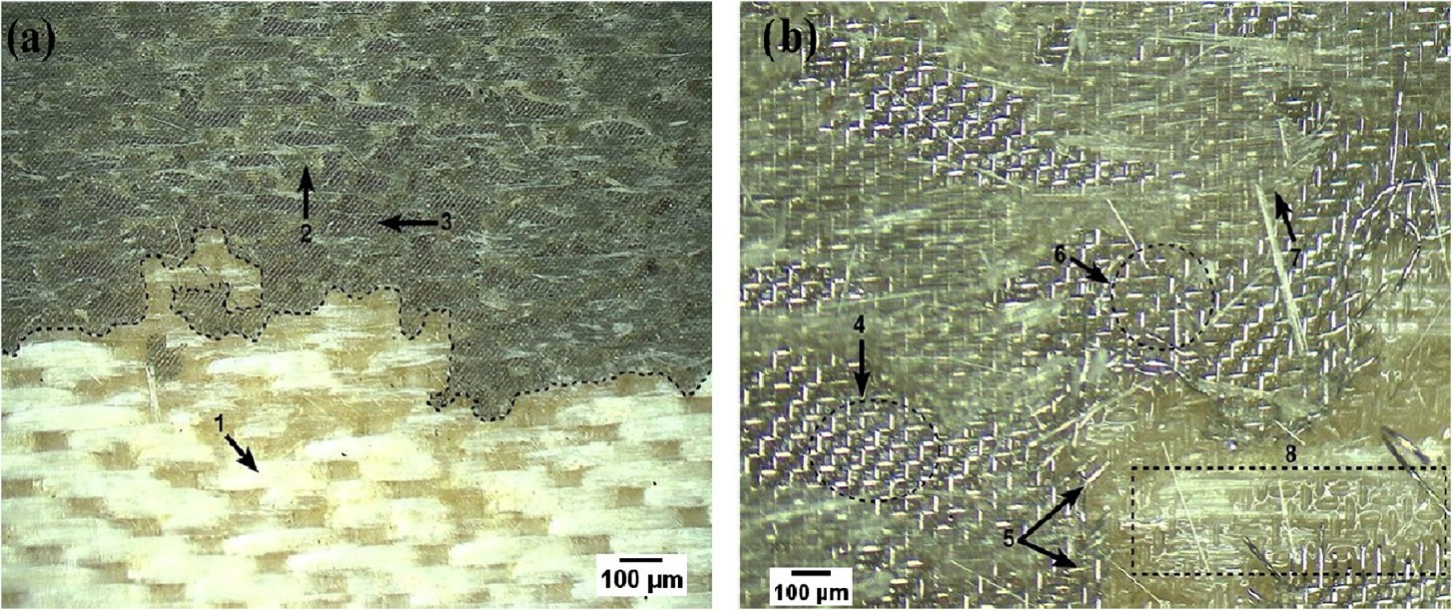
Fractured sample without plasma treatment. Magnifications of 9× (a) and 40× (b).

Figure 19a reveals regions of exposed fibers (1), indicating polymer matrix pull-out. A defined boundary (dotted line) between this region and the HE shows that the fracture occurred via the removal of the lower composite’s matrix, which remained adhered to the HE. Variations in the interaction with the upper composite are also observed: areas where the resin impregnated and was detached from the HE (2) alternates with regions of poor impregnation (3), evidencing irregular adhesion.

At higher magnification (Figure 19b), morphological details become evident. Zones with shallow embedding of the HE wires in the resin (4), areas with metal wire rupture (5), and regions where the wires are significantly deeper within the polymer (6) are identified. This latter aspect is fundamental as greater embedment depth increases the filling of gaps by the resin, enhancing mechanical interlocking and, consequently, joint strength. The presence of voids or resin starvation (3) constitutes sites that facilitate crack initiation and propagation.^15,63,64^ Additionally, plastic deformation is noted in the polymer matrix overlying mesh (7), resulting in its fracture and detachment. This mechanism is more pronounced in the lower composite (8), where intralaminar fracture with matrix pull-out and the imprint of the metal mesh on the surface (dashed region) is observed, as reported in similar studies.^62^

For the material welded with HMDSO (Figure 20), an amplification of the observed mechanisms is noted. The fracture morphology, while still exhibiting matrix pull-out and intralaminar fracture, reveals more extensive polymer coverage over the HE and significantly deeper embedding of the wires in the lower matrix.

**Figure 20:**
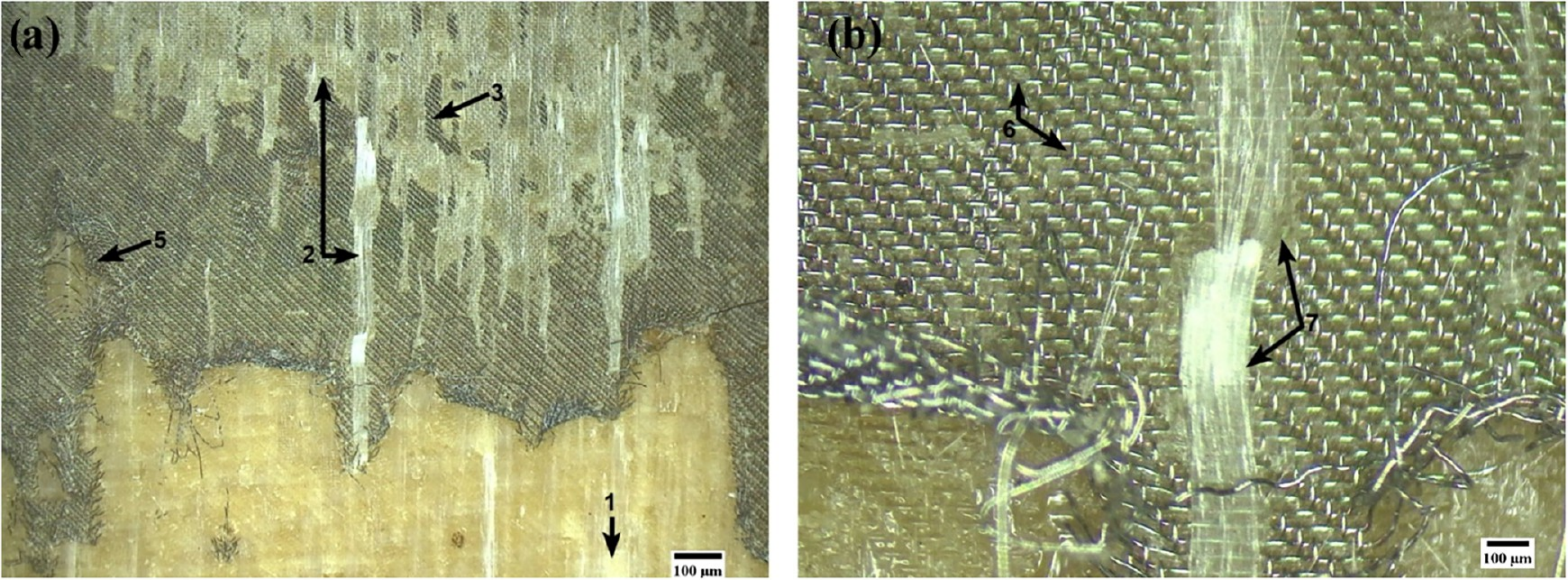
Fractured sample with HMDSO deposition. Magnifications of 9× (a) and 40× (b).

These characteristics indicate a decisive improvement in resin flow and penetration during welding, which fills the mesh gaps more effectively. This effect is attributed to the role of the HMDSO film as a rheological facilitator rather than a chemical coupling agent. Although the methyl-rich surface exhibits a low chemical affinity with the matrix, the hydrophobic configuration of the HMDSO film reduces interfacial friction between the molten polymer and the metal wire. This “lubricating effect” facilitates the flow of the high-viscosity softened resin through the mesh apertures under pressure. Consequently, the resin is able to fully envelop the wires and fill the mesh voids, promoting superior macroscopic mechanical interlocking. The resulting increase in joint strength is therefore driven by the larger effective contact area and the formation of resin “rivets” through the mesh, rather than chemical bonding.

The O_2_ plasma post-treatment (Figure 21) promoted a qualitative evolution in adhesion. The functionalized surface presented an expansion and accentuation of the regions of resin coverage and penetration into the HE. This morphology is directly correlated to the selective oxidation of the film, which introduced carboxylic and hydroxyl groups as evidenced by XPS. The conversion to a hydrophilic and chemically active surface increased the surface free energy, creating a thermodynamic driving force for enhanced wettability. This allowed the molten PEI matrix to establish intimate contact with the HE wires, promoting strong physicochemical interactions (such as hydrogen bonding and van der Waals forces) with the oxidized silicon network (SiO_*x*_).^31,65^ Consequently, the predominant failure mode became cohesive pull-out (matrix + HE), demonstrating that the joint withstood higher loads before fracture compared to the other conditions due to this synergistic effect of mechanical interlocking and secondary chemical bonding.

**Figure 21:**
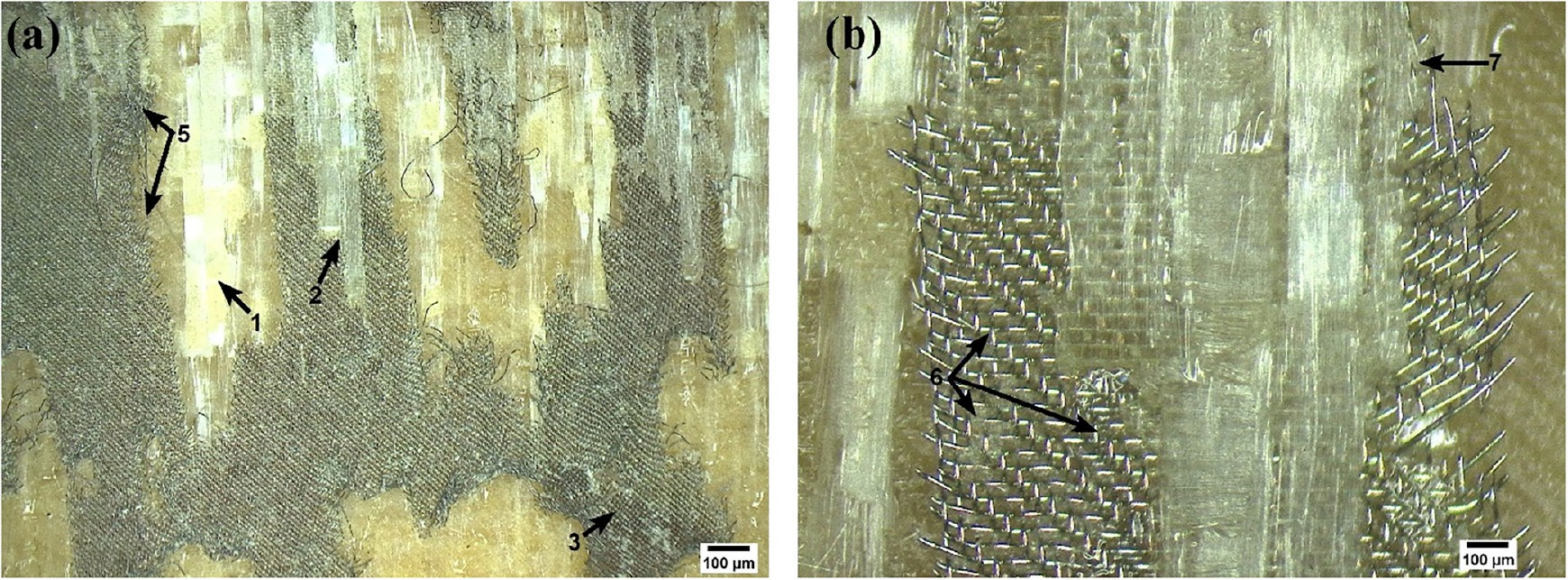
Fractured sample with HMDSO+O_2_ deposition. Magnifications of 9× (a) and 40× (b).

The welded composites exhibited predominantly intralaminar fracture behavior, indicating good interfacial interactions in the matrix/matrix and matrix/HE systems. Evidence such as plastic deformation marks on the polymer and pull-out zones confirms the region subjected to mechanical stress and points to efficient anchoring between the materials.

Detailed fractographic analysis revealed characteristic features that elucidate the rupture mechanisms present under all conditions. River marks—patterns resulting from the coalescence of multiple crack planes converging in the direction of propagation—were observed, along with a low presence of cusps—small structures in the form of inclined platelets, typical of Mode II shear, shown in Figure 22. Large quantities of these cusps may indicate a weak interface, favored by debonding around the fiber, which can lead to cusp formation. The surface also presents matrix abrasion regions and extensive plastic deformation, indicative of the material’s capacity to dissipate energy prior to final failure. The transition between river marks and cusp formation illustrates the crack propagation sequence and corroborates the mixed nature (interlaminar/intralaminar) of the fracture with a higher prevalence of intralaminar fracture. The presence specifically of the intralaminar element points to a welded interface with a good level of adhesion that promoted a cohesive failure in the matrix, reflecting the high quality of the joint and the effectiveness of the applied surface treatment.^44,62,66^

**Figure 22:**
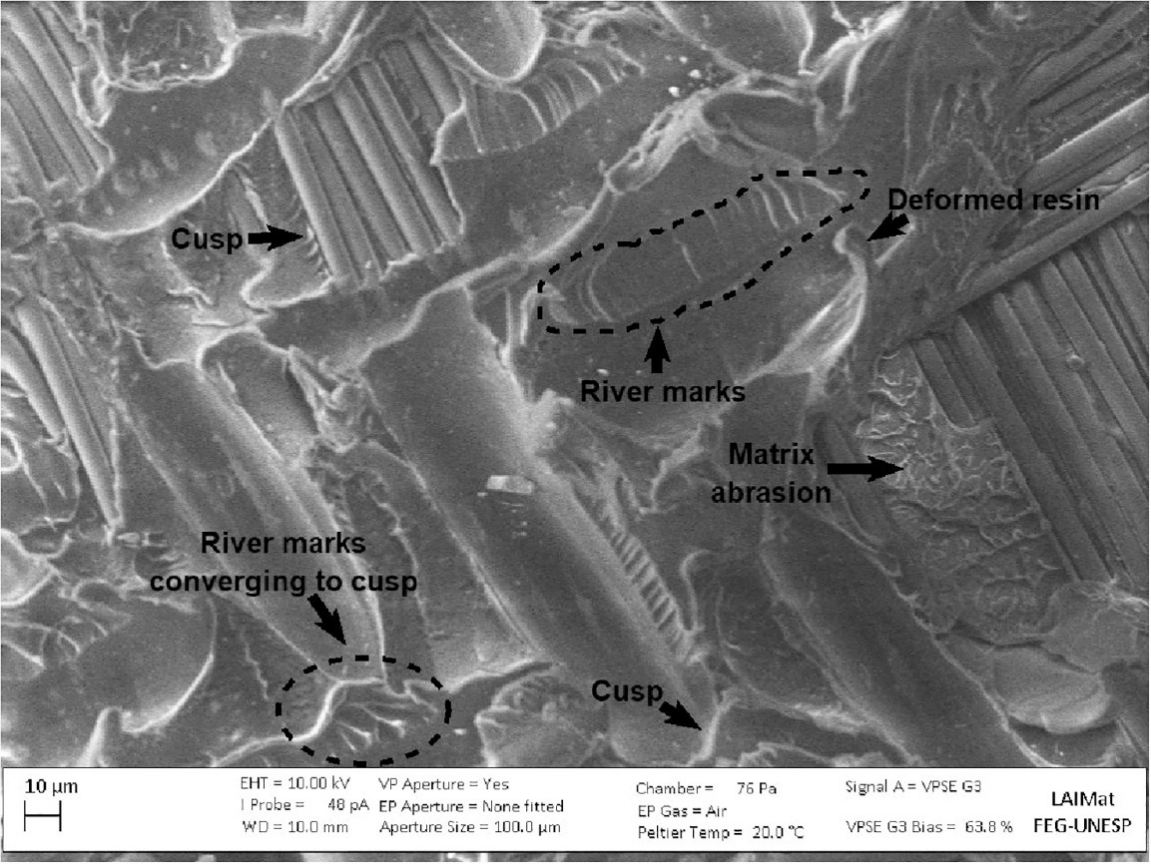
Fractographic features commonly observed in samples.

Nevertheless, fracture features and distinct interaction characteristics were identified for each treatment condition, with their comparison evidenced in Figure 23. In the as-received samples (Figure 23a,b), the fracture behavior is characterized by the presence of river marks and cusps in the matrix, indicative of local plastic deformation during crack propagation. Although there are regions where the matrix remains adhered to the mesh, the absence of severe structural damage to the metallic reinforcement suggests that interfacial failure occurred before the maximum tensile stress of the metal was reached. Adhesion, in this case, relies primarily on mechanical interlocking and natural wettability, with no evidence of chemical bonding.

**Figure 23:**
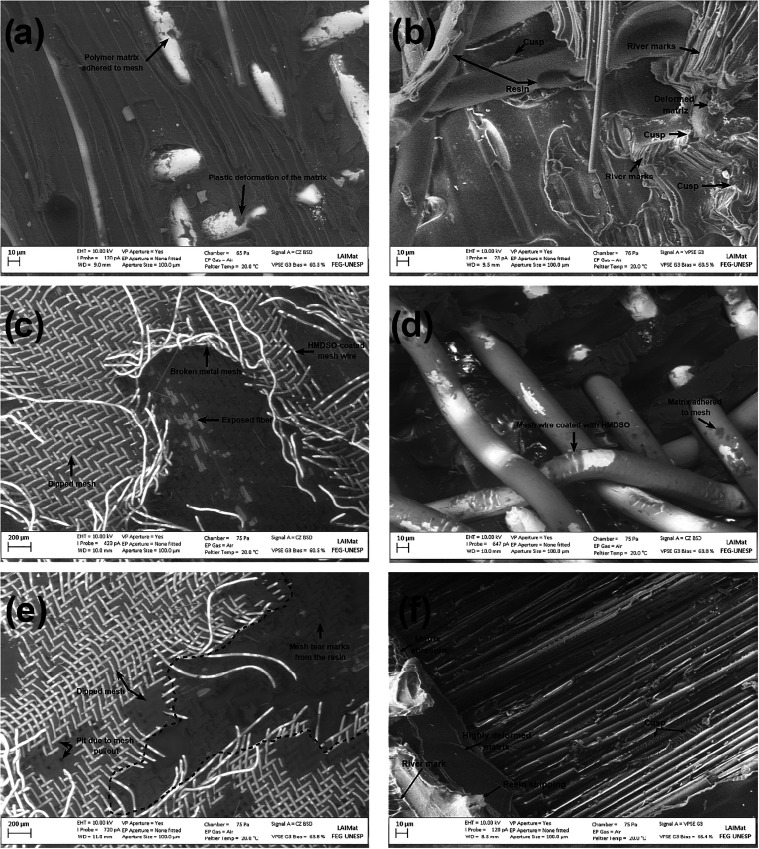
SEM images of materials without treatment (a and b), with HMDSO deposition (c and d), and with HMDSO+O_2_ deposition (e and f).

The samples treated with the HMDSO film (Figure 23c,d) present, as a main feature, the fracture of the metal mesh wires (Figure 23c). This behavior is a clear indication of high interfacial strength, demonstrating that the interface was able to efficiently transfer stress from the matrix to the reinforcement, exceeding the tensile strength of the metal itself, albeit in more localized regions. Furthermore, a more extensive coverage of the mesh gaps is observed, evidencing the action of HMDSO as a facilitator of softened matrix flow during welding, resulting in a more effective joint.

The subsequent O_2_ plasma treatment on the HMDSO film (Figure 23e,f) led to a significant evolution of the features present in the interfacial morphology. In addition to the gap coverage, a deeper embedding of wires into the matrix and an intensification of the pull-out occurrences are noted. The presence of larger regions with deep matrix and HE wire pull-out suggests that the mechanism of interfacial adhesion strength exceeding the rupture energy of the HE itself occurred here as well, as in the previous situation but presented greater homogeneity in the welded region. This combination of greater penetration, coverage, and pull-out is directly associated with the increased wettability and chemical adsorption promoted by the oxygenated groups introduced by the plasma, which enhances the interactions between the functionalized surface and the polymeric matrix.

## 4 Conclusions

The present study demonstrated the efficacy of surface modification of stainless-steel meshes (AISI 304) via PECVD to improve the resistance welding of GF/PEI thermoplastic composites. Both the deposition of HMDSO thin films and their combination with oxygen plasma functionalization proved to be robust strategies to overcome interfacial incompatibility at the metal–polymer junction.

The Taguchi L9 experimental design allowed identification of the power as the most influential parameter on deposition quality, followed by process time, while composition presented a marginal effect. The optimized condition (30 W, 60% HMDSO, and 30 min) produced films with adequate cross-linking, avoiding both excessive degradation and the formation of poorly adherent oligomers observed at high precursor concentrations.

FTIR and XPS characterizations elucidated the distinct adhesion mechanisms for each condition. Films with hydrophobic characteristics (HMDSO only) improved adhesion primarily through enhanced mechanical interlocking. The hydrophobic surface acted by reducing interfacial friction, which facilitated the rheological flow of the softened matrix through the mesh gaps, allowing for better void filling without chemical bonding. Conversely, the O_2_ plasma post-treatment was crucial for altering the surface nature, promoting the oxidation of the organosilane layer and introducing oxygenated functional groups (Si–OH, Si–O–C). This chemical alteration converted the surface from hydrophobic to hydrophilic, increasing surface energy and driving thermodynamic wettability. This ensured intimate contact and promoted strong physicochemical interactions (such as hydrogen bonding) between the matrix and HE, enhancing joint strength through a synergistic effect of mechanical anchoring and chemical affinity.

The welded joints showed a significant increase for both treatment conditions, with the dual treatment (HMDSO+O_2_ Plasma) presenting the best performance with a significant 48% increase in shear strength (SLSS) compared to the control joints (untreated). This condition reached average values exceeding 15.44 MPa and presented fractographic features characteristic of intralaminar failure, greater coverage and embedding of HE wires by the matrix, and pull-out regions. In addition to the strength gain, the treatment reduced dispersion results, indicating greater reproducibility and reliability of the joining process.

As future perspectives, the investigation of the behavior of these joints under fatigue loading and after hygrothermal aging is suggested in order to validate the durability of the modified interface under real service conditions. Furthermore, numerical modeling of the weld zone, considering the new interfacial properties obtained, may offer valuable insights into the design of complex structural components.
